# Comparative analysis of the anthelmintic efficacy of European heather extracts on *Teladorsagia circumcincta* and *Trichostrongylus colubriformis* egg hatching and larval motility

**DOI:** 10.1186/s13071-022-05531-0

**Published:** 2022-11-04

**Authors:** Francesca Shepherd, Caroline Chylinski, Michael R. Hutchings, Joana Lima, Ross Davidson, Robert Kelly, Alastair Macrae, Juha-Pekka Salminen, Marica T. Engström, Veronika Maurer, Håvard Steinshamn, Susanne Fittje, Angela Morell Perez, Rocío Rosa García, Spiridoula Athanasiadou

**Affiliations:** 1 Scotland’s Rural College (SRUC), Easter Bush Campus, Midlothian, UK; 2 Royal (Dick) School of Veterinary Studies [R(D)SVS]–the Roslin Institute, University of Edinburgh, Easter Bush Campus, Midlothian, UK; 3 Department of Chemistry, University of Turku, Turku, Finland; 4 Research Institute of Organic Agriculture (FiBL), Frick, Switzerland; 5 Norwegian Institute of Bioeconomy Research (NIBIO), Tingvoll, Norway; 6 Naturland Association for Organic Agriculture, Gräfelfing, Germany; 7 Asociación Valor Ecologico (ECOVALIA), CAAE Association, Seville, Spain; 8 Agrifood Research and Development Regional Service (SERIDA), Villaviciosa, Asturias Spain

**Keywords:** *Teladorsagia circumcincta*, *Trichostrongylus colubriformis*, Gastrointestinal nematode, Proanthocyanidin, Condensed tannins, Anthelmintic, Plant extracts

## Abstract

**Background:**

Gastrointestinal nematode (GIN) control is traditionally achieved with the use of anthelmintic drugs, however due to regulations in organic farming and the rise in anthelmintic resistance, alternatives are sought after. A promising alternative is the use of bioactive plant feeding due to the presence of plant secondary metabolites (PSMs) such as proanthocyanidins (PAs). This study focussed on the perennial shrub heather (*Ericaceae* family), a plant rich in PAs, highly abundant across Europe and with previously demonstrated anthelmintic potential.

**Methods:**

In vitro assays were used to investigate heather’s anthelmintic efficacy against egg hatching and larval motility. Heather samples were collected from five European countries across two seasons, and extracts were tested against two GIN species: *Teladorsagia circumcincta* and *Trichostrongylus colubriformis*. Polyphenol group-specific ultraperformance liquid chromatography-tandem mass spectrometry analysis was performed to identify relevant polyphenol subgroups present, including the PA concentration and size and ratio of the subunits. Partial least squares analysis was performed to associate efficacy with variation in PSM composition.

**Results:**

Heather extracts reduced egg hatching of both GIN species in a dose-dependent manner by up to 100%, while three extracts at the highest concentration (10 mg/ml) reduced larval motility to levels that were not significantly different from dead larvae controls. PAs, particularly the procyanidin type, and flavonol derivatives were associated with anthelmintic activity, and the particular subgroup of polyphenols associated with the efficacy was dependent on the GIN species and life stage.

**Conclusions:**

Our results provide in vitro evidence that heather, a widely available plant often managed as a weed in grazing systems, has anthelmintic properties attributed to various groups of PSMs and could contribute to sustainable GIN control in ruminant production systems across Europe.

**Graphical Abstract:**

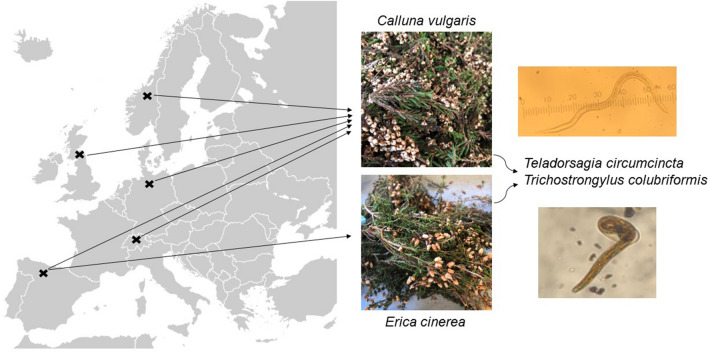

**Supplementary Information:**

The online version contains supplementary material available at 10.1186/s13071-022-05531-0.

## Background

Parasitism has been identified as the main health challenge for ruminants [1], with gastrointestinal nematodes (GIN) demonstrating high farm prevalence across Europe, which can lead to considerable production costs and animal welfare penalties. Indeed, economic losses from parasitism have been shown on at least 50% of farms globally [2, 3], with GIN costing ruminant farming in Europe an average of €686 million per year [4]. The prevalence of GIN in some European countries can be up to 100% on sheep farms [5, 6], and a global review reported that GIN infections in sheep resulted in negative production effects in 86% of the studies reviewed [7]. High GIN prevalence is also shown on cattle farms, as a UK study found 89% of cattle in English and Welsh abattoirs had evidence of GIN [8]. With the global rise of anthelmintic resistance over the past 50 years [9] and a move towards organic and low-input farming systems [10], alternative GIN control strategies are needed to replace, or complement, the use of synthetic anthelmintics. There is an increasing interest in the use of bioactive plant feeding as a sustainable GIN control method, in particular for small ruminants. Numerous in vitro studies have demonstrated the anthelmintic potential of plant extracts rich in active compounds on a range of GIN life stages. The anthelmintic activity of many bioactive plants is often attributed to proanthocyanidins (PAs; synonym: condensed tannins) [11–13], although other plant secondary metabolites (PSMs; also known as plant specialised metabolites), including hydrolysable tannins and flavonoids, have been implicated [14, 15]. Furthermore, studies have demonstrated that the anthelmintic activity of bioactive plants varies across different GIN species, suggesting that the efficacy may be GIN species specific [13, 16, 17].

The perennial shrub heather is highly abundant across European countries, indicating the plant’s amenability as a forage, and is rich in PAs [12]. Previous studies have shown that Spanish heather extracts have in vitro anthelmintic effects on GIN exsheathment of infective third-stage larvae (L3), adult motility and the inhibition of egg hatching [18, 19]. The relative chemical composition of heather is known to vary between geographic location, time of year and species [11, 20–22]. For example, total phenolics content in heather measured in different studies ranged from 94 to 118 g tannic acid equivalent (TAE) per kilogram dry matter (DM) of heather [23–25], whereas total tannins content varied from 64 to 86 g TAE/kg DM [11, 23–25]. The impact of these variations in the chemical composition of heather on the variation in the anthelmintic efficacy observed has not yet been explored. An investigation of this relationship is important to determine whether heather can play a role as an alternative strategy for GIN control across European farming systems.

The objective of this study was to conduct a comparative analysis of the anthelmintic efficacy of *Calluna vulgaris* extract from samples collected in five European countries where *C. vulgaris* is native, across two seasons, and tested against two GIN species. As there are two equally prevalent and native species in Spain, in addition to *C. vulgaris, Erica cinerea* samples were also collected from Spain. It was hypothesised that the anthelmintic activity of the heather extracts would be driven by their tannin content (PAs and hydrolysable tannins), as this is generally influenced by environmental factors, including the country, season (local conditions) and plant species. It was also hypothesised that the anthelmintic activity of heather extracts will vary across GIN species and life stages.

## Methods

### Heather collection and drying process

Heather samples from five European countries, namely Germany, Norway, Spain, Switzerland and the UK, were collected and sent to Scotland’s Rural College (SRUC) in Edinburgh for drying and extraction processes. Heather winter samples were harvested between January and March (except for Switzerland, where due to snow at the site, the collection was completed in April) and spring samples from March to May. The same collection and processing protocols were followed at each location (for details, see Additional file 1: Text S1, Additional file 1: Figure S1). Details of collected heather samples are shown in Table 1, with environmental conditions and GPS co-ordinates at the sites of heather collection included in Additional file 2: Table S1. Upon receipt at SRUC, samples were dried at 25 °C until dry weight was reached, as determined by consecutive weighing. The dried samples were then blended into powder, wrapped in polyethene bags and aluminium foil to avoid exposure to light and stored at − 70 °C until further analysis.

**Table 1:** Details of collected heather samples

Sample no.	Country	Site name	Heather species	Date sampled	Season
1	UK	Castle Law	*Calluna vulgaris*	25 January 2019	Winter
2	UK	Castle Law	*C. vulgaris*	22 May 2019	Spring
3	Germany	Schneverdingen	*C. vulgaris*	15 January 2019	Winter
4	Germany	Schneverdingen	*C. vulgaris*	22 May 2019	Spring
5	Norway	Smøla	*C. vulgaris*	15 January 2019	Winter
6	Norway	Smøla	*C. vulgaris*	20 May 2019	Spring
7	Switzerland	Oberer Hummel	*C. vulgaris*	04 April 2019	Winter
8	Switzerland	Oberer Hummel	*C. vulgaris*	23 May 2019	Spring
9	Spain	Illano	*C. vulgaris*	15 January 2019	Winter
10	Spain	Illano	*C. vulgaris*	14 May 2019	Spring
11	Spain	Illano	*Erica cinerea*	15 January 2019	Winter
12	Spain	Illano	*E. cinerea*	14 May 2019	Spring

### Heather extraction for in vitro assays and chemical analysis

The extraction method for this protocol was adapted from Gutu [26]. Briefly, 10 g of powdered heather was chemically macerated in 100 ml of acetone/water solution (70:30, v/v) [27]. The solutions were continuously stirred on a magnetic stirrer at room temperature for 72 h [28] and then filtered through four layers of miracloth (Calbiochem®). The filtrate was centrifuged for 3 min at 4500 rpm and dried twice in a rotary evaporator to remove all water and acetone. Extracts were stored at − 20 °C until their use in in vitro testing and chemical analysis.

The methods used to chemically analyse the heather extracts are described by Malisch et al. [14]. Tannins have been repeatedly shown to contribute to the antiparasitic effects of plants and, in addition, flavonols have been shown to positively affect the antiparasitic effect of tannins [29]. For these reasons we focussed on these polyphenol types by using polyphenol group-specific ultraperformance liquid chromatography-tandem mass spectrometry (UPLC-MS/MS) analysis for their quantification [30]. The extracts were lyophilised and dissolved in 1 ml of Milli-Q purified water, vortexed for 5 min and filtered through a 0.20-µm polytetrafluorethylene (PTFE) filter into UPLC vials. The sample was 1:5 diluted with water and analysed according to Engström et al. [31, 32] on an Acquity UPLC system (Waters Corp., Milford, MA, USA), interfaced to a Xevo TQ triple-quadrupole mass spectrometer with electrospray ionisation (Waters Corp.).

### Gastrointestinal nematodes

The GIN species used in this study are two of the most prevalent ovine GIN across European countries [5, 6, 33]: *Teladorsagia circumcincta* (abomasal) and *Trichostrongylus colubriformis* (intestinal). The GIN infective larvae were originally obtained from Moredun Research Institute, Scotland (*T. circumcincta*) and Toulouse Veterinary School, France (*T. colubriformis*) to infect donor sheep at SRUC. In total, six donor sheep were mono-specifically infected, four with 15,000 *T. circumcincta* L3 and two with 15,000 *T. colubriformis* L3, and these animals produced all the eggs and L3 used in the experiments described below.

### Egg hatch assay

Fresh faecal samples were collected from the rectum of donor sheep, and the eggs were isolated with a flotation technique, previously described by Christie and Jackson [34], using a 1.2 g/ml sodium chloride solution. The eggs were then recovered in a water suspension and pooled within species; approximately 100–200 eggs were added into each well of a 96-well plate. For the stock solution of heather extract, 50 mg of extracted heather was dissolved in 100 µl of a 50% dimethyl sulfoxide (DMSO) solution, along with 25% phosphate-buffered saline (PBS) and 25% distilled water (dH_2_O). Serial dilutions of heather extracts were carried out before 6 µl of freshly prepared heather extract was then added to each well (*n* = 3); additional water was added if needed to make up a total volume of 300 µl. This resulted in final heather extract concentrations of 0.625, 1.25, 2.5, 5 and 10 mg/ml, respectively. Negative control wells (*n* = 3) contained the same volume of egg suspension together with 6 µl of 50% DMSO solution without the heather extract. The final DMSO concentrations were 1% per well for both treatment and control wells, which has previously been shown to be well tolerated by parasites [35, 36]. The assay was set up at room temperature (22 °C), and hatching was stopped at the end of a 48-h period with the addition of 20 µl of Lugol’s reagent to each well. The number of unhatched eggs and first-stage larvae (L1) per well were counted under an inverted microscope. The percentage of eggs hatched was calculated using the following equation:

$${\text{Egg}}\,{\text{hatching}}\;\% = \frac{{{\text{Number}}\;{\text{of}}\;{\text{L}}1}}{{{\text{Total}}\;{\text{number}}\;{\text{of}}\;{\text{unhatched}}\;{\text{eggs}}\;{\text{and}}\;{\text{L}}1}} \times 100$$

#### Polyvinyl polypyrrolidone incubation

To investigate the contribution of tannins on the anthelmintic activity observed, a second set of egg hatch assays (EHA) was carried out using only the highest concentration of heather extract (10 mg/ml) which had been incubated with polyvinyl polypyrrolidone (PVPP). PVPP is an agent which can form complexes with tannins and thus remove their effects. Extracts were incubated overnight with PVPP at a 1:10 ratio as described in Zabré et al. [37], then centrifuged at 4500 rpm for 3 min; the supernatant was used as the ‘PVPP extract’. As previously described, 100–200 eggs were then placed in the wells with extracts incubated with or without PVPP; non-heather controls were also included (6 µl of 50% DMSO solution) that had also been incubated with or without PVPP at a total volume of 300 µl per well (1% DMSO per well). Treatments were carried out in triplicate and as previously described; hatching was stopped at the end of 48 h with the addition of 20 µl of Lugol’s reagent to each well.

### Larval motility assay

The GIN L3 were recovered from faecal samples of the donor sheep after a 10-day incubation period at 20 °C, using the Baermann technique and stored at 4 °C before use in the assay for a maximum duration of 3 months. Real-time cell analysis (RTCA) on the xCELLigence Real Time Cell Analyser (ACEA Biosciences, Agilent Technologies, Santa Clara, CA, USA) was used to monitor larval motility. In this system, movement is detected based on electrical impedance-based signals via interdigitated microelectrodes integrated in the bottom of tissue culture E-plates (Agilent Technologies). This method has previously been used for anthelmintic drug screening and resistance monitoring [38] and modified by Athanasiadou et al. [39] to monitor and quantify plant extract efficacy. A total of 3000 L3 were placed into each well of the E-plate, and readings were taken every 15 s for 24 h to provide a baseline motility reading. After 24 h, 4 µl of the heather extracts was added into each well, making a final reaction volume of 200 µl and final DM heather extract concentration of 10 mg/ml, to match the highest concentration used in the EHA. A series of triplicate control wells were included: (i) 3000 dead L3 (previously placed for 30 min in Milton® sterilising fluid; Proctor & Gamble, Cincinnati, OH, USA); ii) 3000 L3 that were maintained alive throughout; and (iii) two sets of technical control wells without L3, but containing instead a 50% PBS solution. All control wells, except for one set of technical controls which measured the signal of solely heather extract, received 4 µl of 50% PBS after the initial 24 h, instead of heather extract. The same stock solution of heather extract which was dissolved in 50% DMSO for the EHA was used here, so additional control plates were run to demonstrate that DMSO and PBS controls had no impact on larval motility. Larval motility was monitored for a further 24-h period post heather extract or 50% PBS addition. Four experiments were run, two for *T. circumcincta* and two for *T. colubriformis*, and within each GIN species; one experiment was set to test the impact of *C. vulgaris* extracts as affected by country and season, and the other to test the impact of heather species and season of the Spanish samples.

### Statistical analysis

Principal component analysis (PCA) was performed to summarise the variation in the polyphenol subgroup concentrations in each heather extract; PCA plots were produced using the devtools [40] and ggbiplot [41] packages in R (v 4.1.0).

#### Egg hatch assay

A multivariate analysis of variance (ANOVA) test with a Fisher’s protected least significant difference (LSD) multiple comparison correction was used to conduct a comparative analysis on the impact of heather extracts on egg hatching. The aim of this test was to analyse the effect of the season collected, country of origin, GIN species tested on and concentration of *C. vulgaris* extract on egg hatching of *T. circumcincta* and *T. colubriformis*, respectively. ANOVA was carried out to determine the impact of heather species from the Spanish samples, as well as the PVPP impact. The lethal dose of extract that inhibited 50% of egg hatching (LD_50_) was calculated using probit analysis. All tests were carried out using Genstat® 16th edn (2019; VSN International Ltd.).

#### Larval motility assay

Impedance data were processed on R (v 4.1.0) where the variability of the cell index was converted to the motility index [39]. Motility index values for 5 h prior to the addition of the extract and the last 5 h after the addition of the extract were averaged for each well and then analysed using Genstat® 16th edn (2019; VSN International). This time window (at the end of each 24-h period) was chosen for analyses so the extract had settled and was not contributing to motility readings, and to allow time for the extract to have an impact. A one-way ANOVA was used to analyse the impact of the heather extract on larvae motility, whereas the average motility prior to the addition of the extract was used as a covariate. If the impact of the covariate was not significant (*P* > 0.05), it was removed from the model to prevent data being skewed. The null hypothesis was that the motility of larvae exposed to heather extract would significantly differ (*P* < 0.05) from that of dead larvae, but not from that of alive control larvae (*P* > 0.05). An additional two-way ANOVA was carried out to associate larval motility with treatment factors: season, country and heather species.

To explore which polyphenol subgroup combinations were more strongly associated with anthelmintic activity (LD_50_ from EHA, or motility values), we calculated partial least squares (PLS) regressions with the mixOmics package [42] in R (v 4.1.0). PLS models are suitable for this analysis because the correlations between the different explanatory variables (i.e. polyphenol subgroups) are considered. The number of latent variables used were determined by ‘leave-one-out’ cross-validation. Additionally, PLS models provide a variable importance in projection (VIP) score for each explanatory variable, allowing for the identification of explanatory variables more closely associated with variation in the dependent variable. Regression coefficients that indicate strength and direction of associations were also produced.

## Results

### Large variation in polyphenol content was evident in heather samples

The results of the chemical analysis of the heather extracts are shown in Table 2. The polyphenol subgroups quantified in the analyses were PAs (procyanidin [PC] and prodelphinidin [PD] subunits), hydrolysable tannins (galloyl and hexahydroxydiphenoyl [HHDP] derivatives), flavonol derivatives (kaempferol, quercetin and myricetin derivatives) and quinic acid derivatives (e.g. caffeoyl quinic acids). The PAs in the studied extracts were dominated by PC subunits, with the Spanish extracts showing the highest PD:PC ratio. For many of the studied extracts, samples collected in winter had higher concentrations of the quantified polyphenol subgroups, with the exception of the samples collected in Spain. However, extracts from spring samples consistently had a higher mean degree of polymerisation (mDP) of PAs compared to winter samples from the same country.

**Table 2:** Mean concentration of polyphenol subgroups, and the mean degree of polymerisation and subunit ratio of proanthocyanidins detected in the heather extracts used in in vitro tests, obtained from the ultraperformance liquid chromatography-tandem mass spectrometry analysis (*n* = 2)

Country	Season	Heather species	Galloyl derivatives (mg/g)	HHDP derivatives (mg/g)	Quinic acid derivatives (mg/g)	Kaempferol derivatives (mg/g)	Quercetin derivatives (mg/g)	Myricetin derivatives (mg/g)	PC subunits of PA (mg/g)	PD subunits of PA (mg/g)	Total PAs (PC + PD) (mg/g)	mDP of PA
UK	Winter	*Calluna vulgaris*	0.20 (0.01)	0.00 (0.00)	1.33 (0.10)	0.35 (0.02)	0.97 (0.06)	0.00 (0.00)	3.85 (0.37)	0.16 (0.01)	4.01 (0.37)	6.90 (0.00)
	Spring		0.00 (0.00)	0.00 (0.00)	0.90 (0.05)	0.15 (0.01)	0.55 (0.03)	0.00 (0.00)	3.96 (0.30)	0.12 (0.01)	4.07 (0.31)	9.31 (0.10)
Germany	Winter		0.30 (0.01)	0.17 (0.03)	1.31 (0.02)	1.26 (0.02)	1.66 (0.04)	0.11 (0.00)	4.57 (0.11)	0.20 (0.00)	4.77 (0.12)	7.13 (0.01)
	Spring		0.05 (0.05)	0.06 (0.06)	0.51 (0.06)	0.61 (0.04)	0.91 (0.08)	0.00 (0.00)	2.77 (0.17)	0.05 (0.05)	2.82 (0.22)	8.58 (0.06)
Norway	Winter		0.41 (0.00)	0.00 (0.00)	2.17 (0.01)	0.56 (0.01)	1.53 (0.01)	0.19 (0.00)	3.94 (0.02)	0.17 (0.00)	4.12 (0.02)	7.23 (0.03)
	Spring		0.37 (0.04)	0.00 (0.00)	1.87 (0.05)	0.47 (0.01)	1.35 (0.05)	0.18 (0.01)	4.56 (0.21)	0.15 (0.01)	4.71 (0.22)	8.19 (0.11)
Switzerland	Winter		0.30 (0.07)	0.00 (0.00)	2.23 (0.34)	0.97 (0.11)	1.47 (0.11)	0.06 (0.06)	4.43 (0.70)	0.22 (0.03)	4.65 (0.73)	7.37 (0.03)
	Spring		0.20 (0.00)	0.18 (0.01)	0.86 (0.01)	0.52 (0.00)	0.79 (0.00)	0.22 (0.01)	2.82 (0.05)	0.22 (0.00)	3.04 (0.05)	8.05 (0.03)
Spain	Winter		0.22 (0.02)	0.17 (0.00)	1.42 (0.11)	0.62 (0.03)	1.34 (0.10)	0.05 (0.05)	4.41 (0.36)	0.50 (0.04)	4.91 (0.40)	10.6 (0.04)
	Spring		0.36 (0.00)	0.33 (0.01)	1.57 (0.00)	0.82 (0.01)	2.06 (0.03)	0.16 (0.00)	5.79 (0.06)	0.61 (0.00)	6.40 (0.06)	12.2 (0.07)
	Winter	*Erica cinerea*	0.00 (0.00)	0.00 (0.00)	1.29 (0.01)	0.19 (0.00)	0.80 (0.02)	0.12 (0.01)	3.48 (0.10)	0.48 (0.02)	3.96 (0.12)	9.87 (0.06)
	Spring		0.00 (0.00)	0.14 (0.01)	1.47 (0.02)	0.19 (0.00)	0.81 (0.03)	0.14 (0.00)	3.55 (0.05)	0.68 (0.00)	4.24 (0.05)	11.4 (0.04)

Values in table are given as the mean with the standard error (SE) in parentheses

*HHDP* Hexahydroxydiphenoyl,* mDP* mean degree of polymerisation,* PA* proanthocyanidin,* PC* procyanidin, *PD* prodelphinidin

**Fig. 1:**
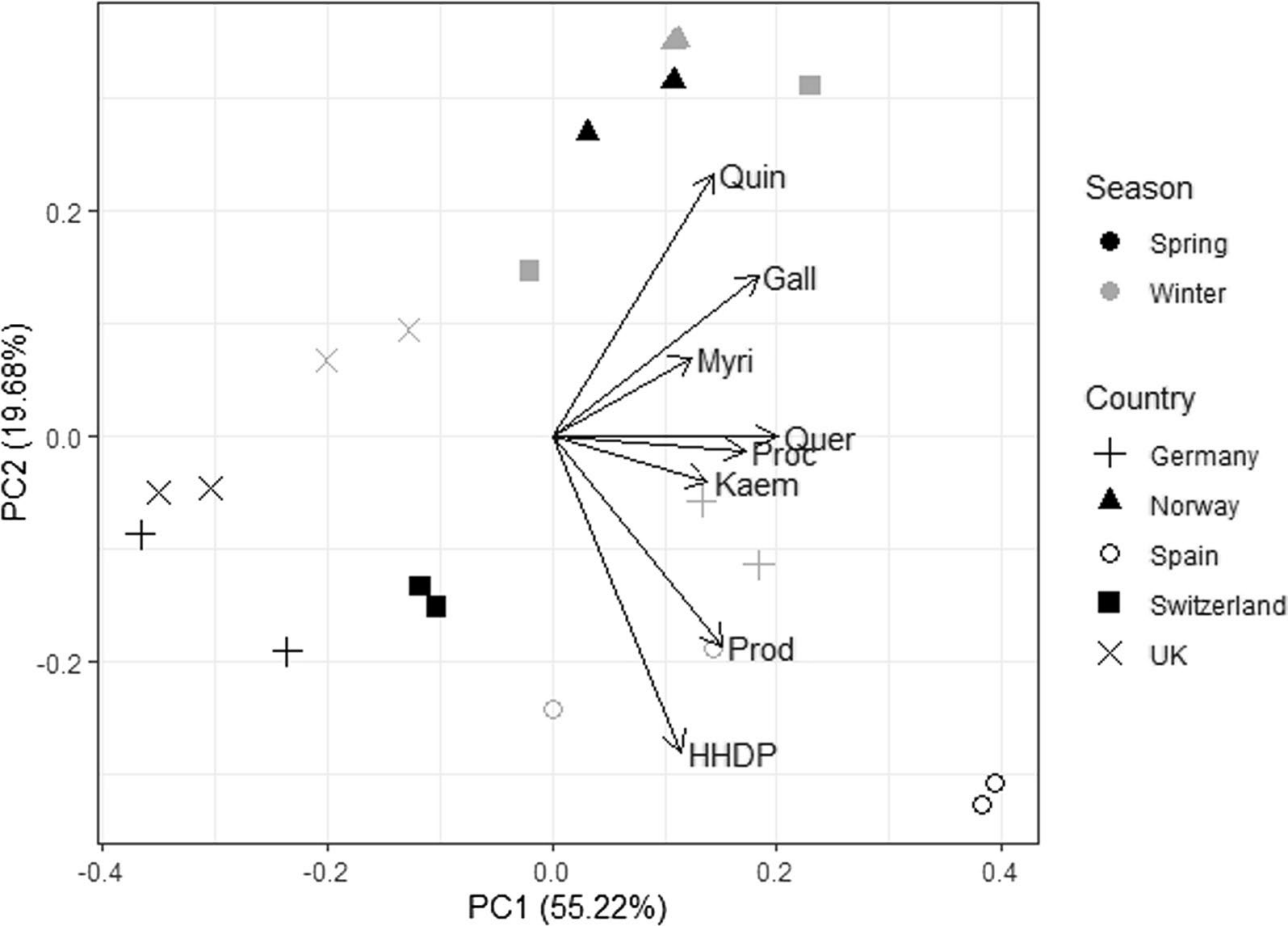
Principal component analysis (PCA) plot of *Calluna vulgaris* samples. Two replicates are shown for each corresponding country and season. The length of the arrow represents the size of the loading, which in turn indicates the explanatory variable’s (i.e. polyphenol subgroup) contribution to the determination of the principal components (PCs). Each polyphenol subgroup is represented by the first four letters of its name. Gall, Galloyl derivatives; HHDP, hexahydroxydiphenoyl derivatives, Kaem, Kaempferol derivatives; Myri, myricetin derivatives; Proc, procyanidin subunits; Prod, prodelphinidin subunits; Quer, quercetin derivatives; Quin, quinic acid derivatives

The first two principal components of the PCA using *C. vulgaris* extracts explained 74.9% of the variation in the concentration of the polyphenol subgroup. There was no significant clustering of samples based on the season of collection or the country of origin, and the two replicates for each extract were similar. The PCA plot for *C. vulgaris* (Fig. 1) demonstrates how heather extracts vary in polyphenol subgroup concentration; for example heather extracts from Spain are seen to have the highest levels of PD subunits and HHDP derivatives, while the contents of quinic acid derivatives were highest in Swiss heather extracts collected in winter and in Norwegian heather extracts collected across both seasons.

**Fig. 2:**
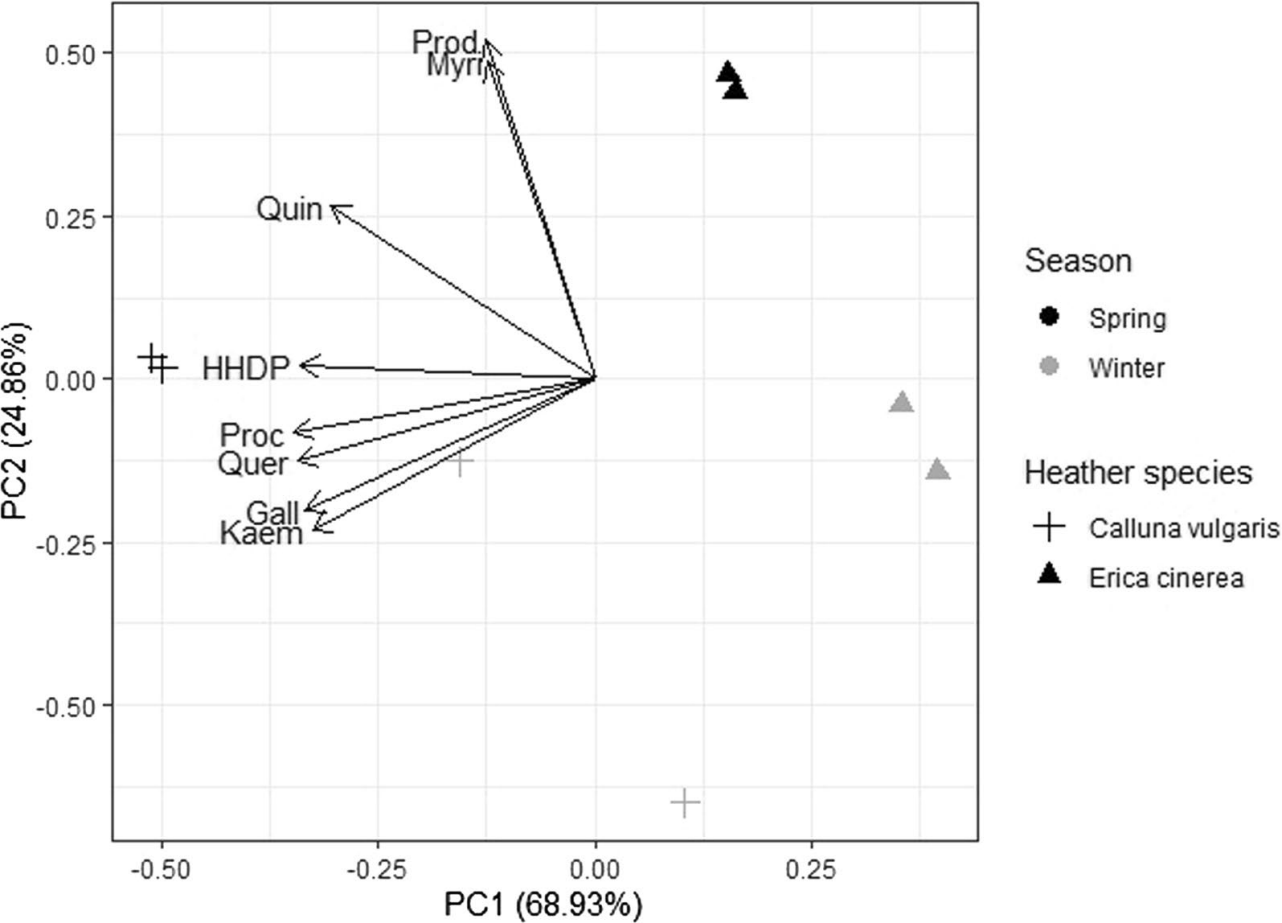
PCA plot of Spanish samples. Two replicates are shown for each corresponding heather species and season. The length of the arrow represents the size of the loading, which in turn indicates the explanatory variable’s (i.e. polyphenol subgroup) contribution to the determination of the PCs. Each polyphenol subgroup is represented by the first four letters of its name (see caption to Fig. 1)

Regarding the comparison of Spanish extracts (Fig. 2), the first two principal components of the PCA explained 93.8% of the variation in polyphenol subgroup concentration. Variations in polyphenol subgroup concentration were observed between the season of collection and the species of the heather extracts. In general, concentrations were higher in *C. vulgaris* extracts compared with *E. cinerea* extracts, although extracts from *E. cinerea* samples collected in spring had higher levels of PD subunits of PAs.

### Heather extracts from the UK and Spain showed the highest efficacy against egg hatching

**Fig. 3:**
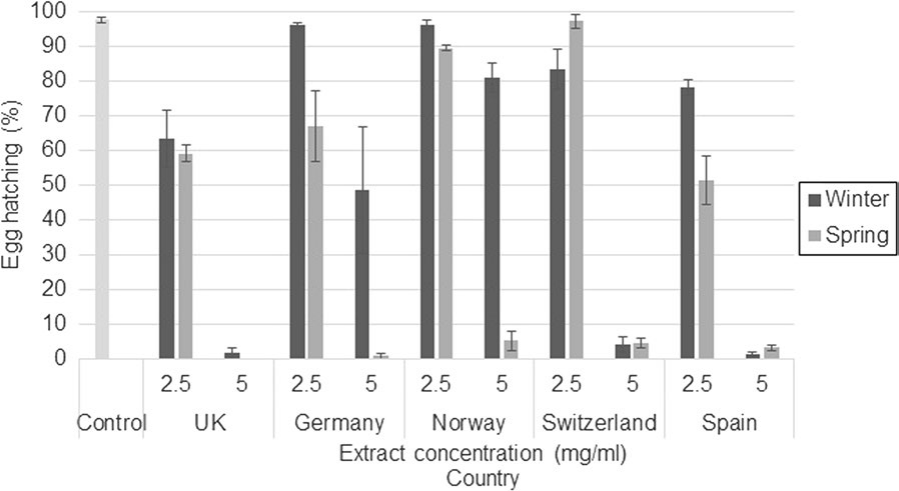
Egg hatching (%) and standard error (SE) bars for *Teladorsagia circumcincta* eggs exposed to 2.5 and 5 mg/ml of *C. vulgaris* extract from each country participating in the study

**Fig. 4:**
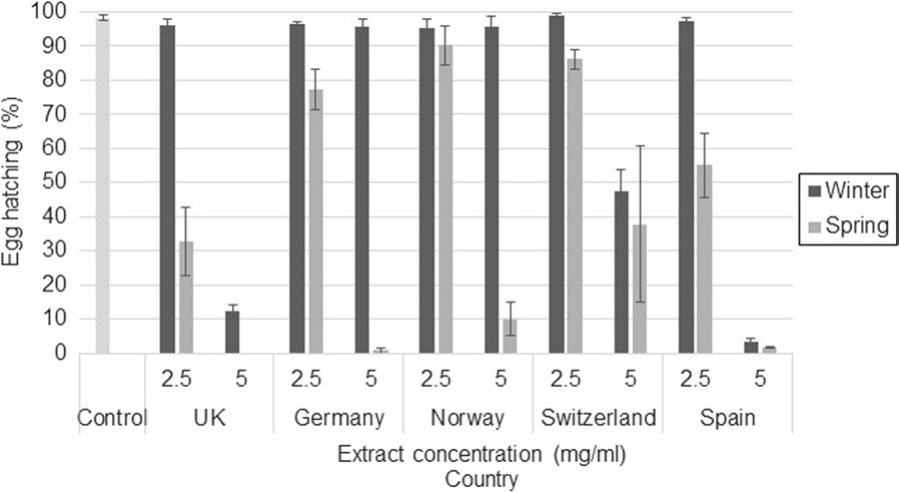
Egg hatching (%) and SE bars for *Trichostrongylus colubriformis* eggs exposed to 2.5 and 5 mg/ml of *C. vulgaris* extract from each country participating in the study

Mean egg hatching in the control wells was 97.9% (standard error [SE]: 0.59) across both GIN species. The concentration of *C. vulgaris* extracts interacted with season, GIN species and country as a four-way interaction (*F*_(16, 196)_ = 4.95, *P* < 0.001). Although at *C. vulgaris* extract concentrations of 0.625, 1.25 (no significant difference from the control) and 10 mg/ml (all significantly different from the control) there was no difference in egg hatching across seasons or countries, concentrations of 2.5 and 5 mg/ml showed that egg hatching in *C. vulgaris* extracts from spring samples was significantly lower than egg hatching in extracts from winter samples. Extracts at 5 mg/ml also showed high variation in egg hatching across countries: at this concentration almost no GIN eggs exposed to extracts from the UK and Spain hatched, which was also true for *T. circumcincta* eggs exposed to Swiss extracts. In contrast, for the remaining countries and for Swiss extracts exposed to *T. colubriformis*, average hatching rates ranged between 23.4% and 47.9%. There was a significant interaction between GIN species and season (*F*_(1, 196)_ = 46.57, *P* < 0.001), with the *T. circumcincta* egg-hatching percentage being lower than that for *T. colubriformis* egg hatching in wells where eggs were incubating in winter extracts, but similar egg hatching for both GIN was shown when eggs were incubated with spring extracts. Graphs showing the interactions are shown in Fig. 3 (*T. circumcincta*) and Fig. 4 (*T. colubriformis*) for *C. vulgaris* extracts. Egg-hatching percentages and* P* values for *C. vulgaris* are shown in Additional file 3: Table S2.

As shown with *C. vulgaris* samples above, only extracts at 2.5 and 5 mg/ml showed variation in anthelmintic efficacy for Spanish samples, and this depended on season, heather species and GIN species as a four-way interaction (*F*_(4, 79)_ = 7.26, *P* < 0.001). Eggs exposed to extracts from winter samples at 2.5 mg/ml did not vary in hatching rate between heather species, whereas the hatching rate for spring samples was lower for eggs exposed to *C. vulgaris* extracts. When exposed to extracts at 5 mg/ml, a very low number of eggs hatched across all wells, except for *T. colubriformis* eggs exposed to *E. cinerea* extracts collected in winter, for which an average egg-hatching rate of 55.1% (SE 7.04) was recorded. Egg-hatching percentages and* P* values for the Spanish samples are shown in Additional file 3: Table S3.

LD_50_ values calculated from the five extract concentrations and three replicates for each heather sample ranged from 2.23 to 8.51 mg/ml, with the LD_50_ value dependent on the season collected, country of origin, heather species and GIN species (Table 3). The lowest LD_50_ values, which are indicative of higher efficacy, were found when testing *C. vulgaris* samples from the UK and Spain that had been collected in spring, and against *T. circumcincta*.

**Table 3:** Lethal dose 50 values for heather extracts (*n* = 3) when tested on *Teladorsagia circumcincta* or *Trichostrongylus colubriformis*

Country	LD_50_ values (mg/ml)			
	*Teladorsagia circumcincta*		*Trichostrongylus colubriformis*	
	Winter	Spring	Winter	Spring
UK	2.77 (0.11)	2.67 (0.11)	3.97 (0.12)	2.23 (0.07)
Germany	5.27 (0.23)	3.17 (0.13)	7.20 (0.24)	3.18 (0.12)
Norway	6.15 (0.23)	4.05 (0.18)	8.51 (0.33)	3.76 (0.13)
Switzerland	3.49 (0.17)	3.86 (0.12)	6.13 (0.25)	4.45 (0.18)
Spain *C. vulgaris*	3.07 (0.12)	2.73 (0.11)	3.85 (0.12)	3.04 (0.13)
Spain *E. cinerea*	3.34 (0.12)	3.53 (0.12)	5.69 (0.24)	3.46 (0.12)

Values in table are given as the mean with the SE in parentheses

*LD*_*50*_ Lethal dose for 50% inhibition

**Fig. 5:**
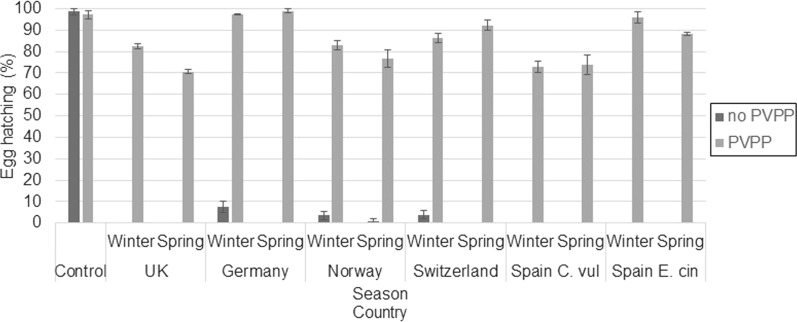
Egg hatching (%) and SE bars of *T. circumcincta* eggs treated with 10 mg/ml of heather extract incubated without PVPP or with PVPP (*n* = 3). PVPP, Polyvinyl polypyrrolidone; C. vul, *Calluna vulgaris*; E. cin, *Erica cinerea*

**Fig. 6:**
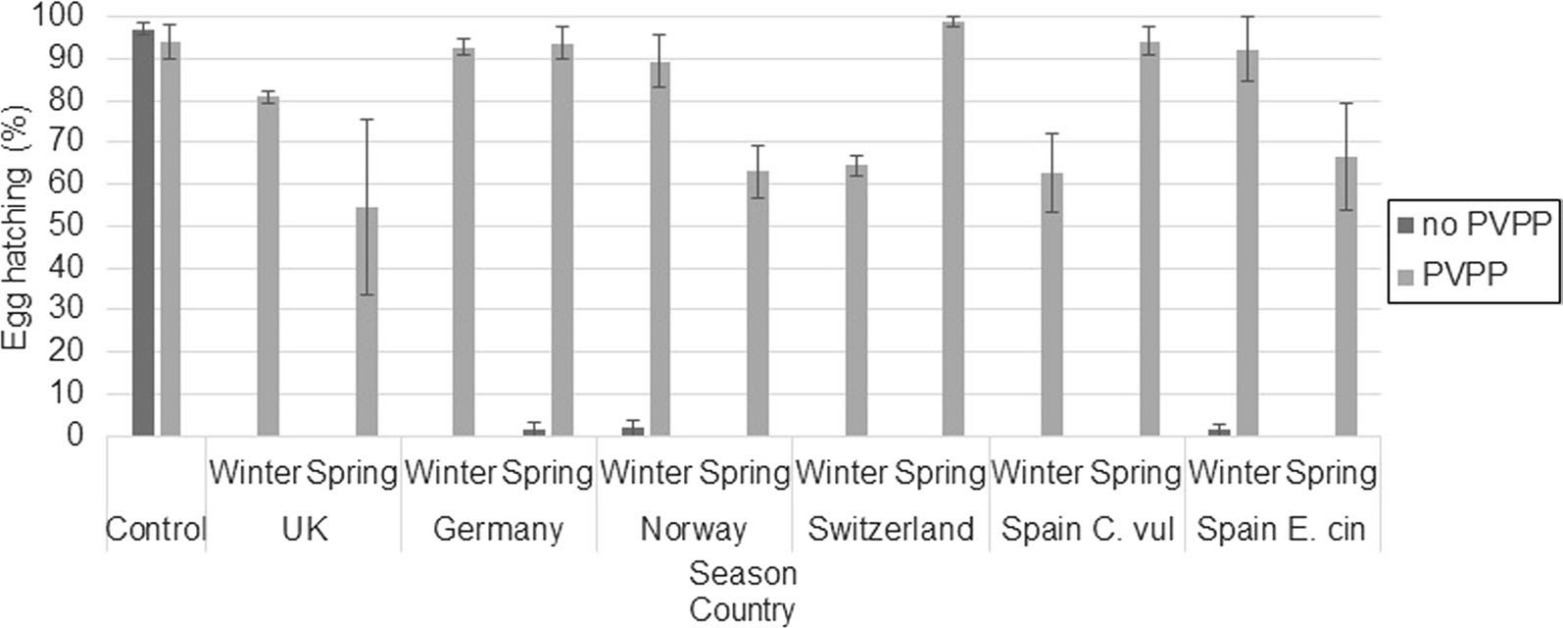
Egg hatching (%) and SE bars of *T. colubriformis* eggs treated with 10 mg/ml of heather extract incubated without PVPP or with PVPP (*n* = 3)

Incubation of extracts with PVPP resulted in higher levels of egg hatching compared to those incubated without PVPP (*F*_(1, 142)_ = 1852.67, *P* < 0.001). Average hatching for eggs incubated in heather extracts at 10 mg/ml without PVPP and with PVPP was 0.87% (SE: 0.27) and 82.2% (SE: 1.87), rerspectively. Egg-hatching percentages for each extract incubated with or without PVPP are shown in Fig. 5 for tests on *T. circumcincta* eggs and in Fig. 6 for tests on * T. colubriformis* eggs.

### Three winter heather extracts reduce L3 motility to the level of dead larvae

**Fig. 7:**
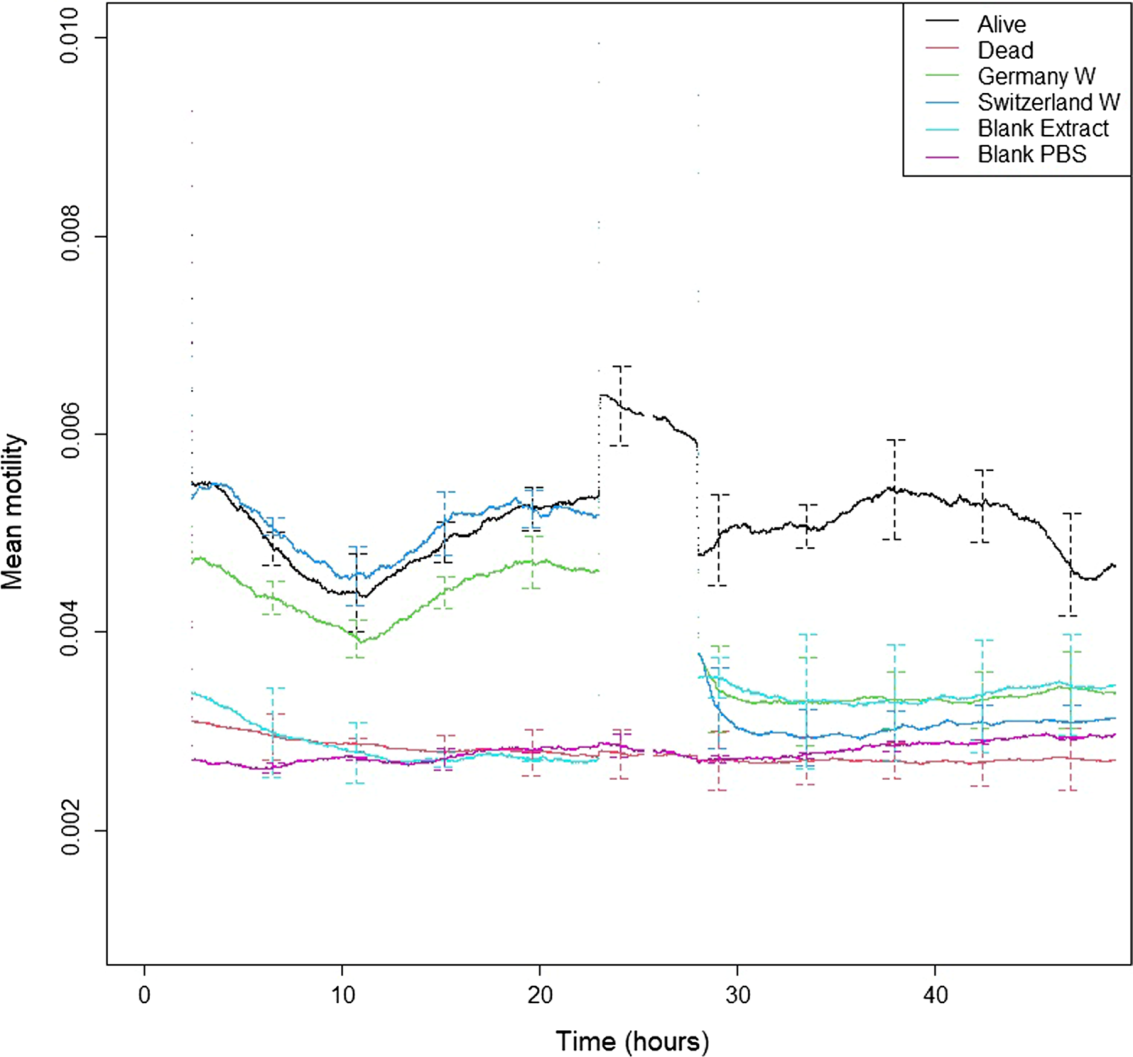
Mean motility readings with SE bars across 48 h for alive, dead and technical controls used in the *T. colubriformis* season × country real-time cell analysis test (*n* = 3). The motility readings for two of the extracts from which the null hypothesis could be rejected are included for reference. PBS, Phosphate-buffered saline; W, extract from samples collected in winter

During the larval motility tests all controls (dead L3, alive L3 and technical controls) behaved as expected (Fig. 7). The variation in motility prior to the addition of* C. vulgaris* extract did not explain any of the variation during the addition of the extracts and, consequently, was not included in the follow-up analyses as a covariate (*P* > 0.05). Also, 1% DMSO did not impact on larval motility (*F*_(1, 4)_ = 0.91, *P* = 0.393), indicating that any changes in larval motility in the treatment wells were solely attributed to the heather extract.

Our null hypothesis, that the motility of larvae exposed to heather extract is significantly different (*P* < 0.05) from that of the dead larvae, but not of the alive control larvae (*P* > 0.05), was rejected on three occasions, namely when *T. circumcincta* larvae were incubated in winter *E. cinerea* samples, and when *T. colubriformis* larvae were incubated in winter samples from Switzerland and Germany, respectively. In many cases, larval motility was lower in the treatment wells compared to the motility of alive control larvae, but it was still significantly different from the motility of the dead controls. The wells containing samples of *C. vulgaris* extracts from the UK and *E. cinerea* extracts from Spain, all collected in spring and tested on *T. colubriformis*, gave motility values that were not different from those of alive controls. These results showed a lack of any anthelmintic effect. Average larval motility values are shown in Table 4.

**Table 4:** Average motility values of larvae for each gastrointestinal nematode species and experiment following incubation with 10 mg/ml of heather extract (*n* = 3)

GIN species	Experiment	Treatments^a^							Controls ^a^		SED
		Season	UK	Germany	Norway	Switzerland	Spain *C. vulgaris*	Spain *E. cinerea*	Alive	Dead	
*T. circumcincta*	Season × Country	Winter	0.0048	0.0046	0.0037	0.0051	0.0044	–	0.0076	0.0013	0.0005
		Spring	0.0055	0.0045	0.0047	0.0050	0.0045	–			
	Species × Season	Winter	–	–	–	–	0.0043	0.0035^b^	0.0073	0.0031	0.0003
		Spring	–	–	–	–	0.0039	0.0045			
*T. colubriformis*	Season × Country	Winter	0.0040	0.0034^b^	0.0036	0.0031^b^	0.0039	-	0.0050	0.0027	0.0004
		Spring	0.0044	0.0040	0.0041	0.0041	0.0041	-			
	Species × Season	Winter	–	–	–	–	0.0039	0.0038	0.0050	0.0027	0.0004
		Spring	–	–	–	–	0.0042	0.0049			

Null hypothesis: the motility of larvae exposed to heather extract will significantly differ (*P* < 0.05) from that of dead larvae, but not from that of alive control larvae (*P* > 0.05)

*GIN* Gastrointestinal nematode, *SED* standard error of difference

^a^Values cannot be compared across different experiments, only within one

^b^Extracts for which the null hypothesis can be rejected

The two-way ANOVA showed that *T. colubriformis* motility was significantly affected by season, with larvae incubated in both *C. vulgaris* (*F*_(1, 20)_ = 11.27, *P* = 0.003) and *E. cinerea* (*F*_(1, 7)_ = 10.30, *P* = 0.015) extracts collected in winter having lower motility than those incubated in the respective extracts collected in spring. For the Spanish extracts, there was a tendency for a significant interaction of season and heather species; *T. colubriformis* larvae incubated in *C. vulgaris* spring extracts and in *E. cinerea* winter extracts had the lowest motility (*F*_(1, 7)_ = 4.29, *P* = 0.077). The motility of *T. circumcincta* larvae tended to be affected by country (*F*_(4, 20)_ = 2.47, *P* = 0.078), with larvae incubated in the extracts from Norway having a lower motility that those incubated in extracts from Switzerland and the UK.

### PAs are not the only polyphenols associated with anthelmintic activity

The PLS analyses were performed to assess the association between the content of the polyphenol subgroup combinations quantified here and the anthelmintic activity of heather extracts, as measured by ovicidal activity (LD_50_). The variation in the polyphenol subgroup contents explained 66.2% and 69.1% of the variation in LD_50_ values for *T. circumcincta* and *T. colubriformis*, respectively. The polyphenol subgroups most importantly associated (VIP ≥ 1) with LD_50_ values for *T. circumcincta* eggs were galloyl and myricetin derivatives, and PC subunits of PAs; for *T. colubriformis* eggs, these were galloyl, quinic acid and kaempferol derivatives, and PC subunits of PAs (Table 5).

**Table 5:** Variable importance of projection and regression coefficient values for egg hatch lethal dose 50 values

Polyphenol subgroup	*T. circumcincta*		*T. colubriformis*	
	VIP^a^	Regression coefficient^b^	VIP^a^	Regression coefficient^b^
Galloyl derivatives	1.18	0.17	1.11	0.03
HHDP derivatives	0.71	− 0.16	0.96	− 0.32
Quinic acid derivatives	0.96	0.19	1.13	0.39
Kaempferol derivatives	0.87	0.25	1.07	0.50
Quercetin derivatives	0.93	0.09	0.92	0.13
Myricetin derivatives	1.31	0.54	0.80	0.38
PC subunits of PA	1.08	− 0.35	1.08	− 0.51
PD subunits of PA	0.83	− 0.20	0.88	0.05

^a^Variable importance of projection (VIPs) represent the importance of each polyphenol subgroup in determining LD_50_ values

^b^The regression coefficient shows the strength and direction of the association

To determine the relationship between the variation in quantified polyphenol subgroup contents and the variation in anthelmintic activity, as measured by the reduction in motility, we included only *C. vulgaris* extracts in the analysis due to an insufficient number of motility readings for the *E. cinerea* samples. PLS analyses showed that the variation in polyphenol subgroup contents explained 41.8% and 85.0% of the variation in motility values for *T. circumcincta* and *T. colubriformis*, respectively. The polyphenol subgroups most importantly associated (VIP ≥ 1) with motility values of *T. circumcincta* L3 were galloyl and quercetin derivatives, and PC subunits of PAs; for *T. colubriformis* L3, these were derivatives of kaempferol and quinic acid (Table 6).

**Table 6:** Variable importance of projection and regression coefficient values for average *C. vulgaris* larval motility values

Polyphenol subgroup	*T. circumcincta*		*T. colubriformis*	
	VIP^a^	Regression coefficient^b^	VIP^a^	Regression coefficient^b^
Galloyl derivatives	1.37	− 0.36	0.96	− 0.15
HHDP derivatives	0.57	0.06	0.79	0.28
Quinic acid derivatives	0.80	− 0.10	1.07	− 0.35
Kaempferol derivatives	0.69	0.01	1.53	− 0.61
Quercetin derivatives	1.28	− 0.31	0.99	− 0.17
Myricetin derivatives	0.79	− 0.16	0.72	0.17
PC subunits of PA	1.39	0.33	0.88	0.13
PD subunits of PA	0.70	0.03	0.85	0.25

^a^VIPs represent the importance of each polyphenol subgroup in determining motility values

^b^The regression coefficient shows the strength and direction of the association

## Discussion

To the best of our knowledge this was the first study in which the anthelmintic efficacy of a shrub native to many European countries was investigated in a comprehensive and comparative manner. Within this study we characterised and quantified the anthelmintic properties of European heather and identified specific types of compounds in heather that can be associated with anthelmintic effects and which of these compounds can be specifically associated with ovicidal or motility inhibition of GIN. We observed variation in the polyphenol content of the extracts originating from the various countries. It is possible that environmental conditions at the collection sites could be contributing to this variation, including light intensity [43], nitrogen (N) addition [44] and grazing [45], as these factors are known to influence polyphenol content in plants. We hypothesised that the observed anthelmintic activity of the heather extracts was driven by the tannin content (PAs and hydrolysable), and this hypothesis was rejected. Variation in tannin content, in particular PC-type PAs of the heather extracts, was associated with some variation in anthelmintic activity, mainly ovicidal activity. However, we showed that other polyphenolic groups, and potentially non-polyphenolic compounds, must be responsible for part of the efficacy observed, especially for inhibition of larval motility. For example, the contents of flavonols such as kaempferol and myricetin derivatives were also closely associated with anthelmintic efficacy. We also hypothesised that the anthelmintic activity of heather extracts varies across GIN species and life stages, which was confirmed, as *T. circumcincta* was more susceptible than *T. colubriformis*, and eggs were more susceptible than larvae.

The observed variation in the combination of polyphenol subgroups quantified in the heather extracts was associated with the observed variation in ovicidal activity (LD_50_), explaining 66–69% of variation in LD_50_ values. The polyphenol subgroups shown to influence the LD_50_ values (VIP ≥ 1) more strongly were galloyl derivatives and PC-type PAs, plus myricetin derivatives for *T. circumcincta* and kaempferol and quinic acid derivatives for *T. colubriformis*. For both GIN species, PC-type PAs showed a moderate negative association with LD_50_, suggesting that PAs and in particular the PC-type are responsible for at least some anthelmintic activity. This may contradict previous evidence showing that PD-type PAs have a stronger association with anthelmintic ability compared to PC-type PAs [16, 46, 47]; however the content of PD-type PAs in the heather extracts was low (average 0.30 mg/g, SE: 0.04), and previous evidence is mainly from tests on larval stages rather than on egg hatching. Our data showed that the addition of PVPP largely reversed egg hatching inhibition, with an average hatching rate reversion of 81.31% (SE: 2.62), although PVPP is not strictly specific at binding PAs, as it can also bind to galloylated flavonoids [48, 49]. Our data are supportive of PAs being responsible for some of the anthelmintic activity observed. Previous studies have demonstrated that some flavonols can enhance anthelmintic activity by acting synergistically with PAs [14, 29, 50]; our data showed that two flavonols we analysed for, myricetin and kaempferol derivatives, had a moderate positive association with LD_50_ (i.e. lower efficacy). As it is unlikely that these flavonols would be improving egg hatching, an antagonistic relationship may be indicated here, which could be attributed to structural differences between the PAs and the other compounds [51].

Unlike the similarity in the associations between polyphenol subgroup content and LD_50_ values for the two GIN species, in the larval mobility test the relative abundance of the polyphenol subgroups explained 85% of the variation in *T. colubriformis* but only 42% of the variation in *T. circumcincta*. This suggests that most of the anthelmintic activity against *T. circumcinta* larvae could be attributed to compounds not quantified here, such as, for example, non-polyphenolic compounds; in contrast, the quantified polyphenol subgroups showed most of the anthelmintic effects against *T. colubriformis* larvae. In the *T. colubriformis* larval mobility tests, the reduced motility was moderately associated with the contents of kaempferol and quinic acid derivatives (VIP ≥ 1). Flavonoid groups, including flavonols, have been previously associated with activity on *Haemonchus contortus* L_3_ exsheathment [50], suggesting that the results of the present study demonstrate that these groups do show activity across other GIN species. As a combination of polyphenol subgroups showed moderate negative associations and VIP ≥ 1, this could indicate additive anthelmintic effects, although any possible synergistic effects cannot be determined by these results. In the *T. circumcincta* larval mobility tests, the groups more strongly associated with motility were galloyl and quercetin derivatives, and PC-type PAs.

Our findings show that susceptibility to the heather extracts was different in life stage in the two GIN species; for example, egg hatching was most affected by heather extracts from the UK and Spain and least affected by extracts from Norway, whereas *T. circumcincta* larval motility was mostly affected by the Norwegian extracts, although still significantly different from the dead control larvae. *Teladorsagia circumcincta* eggs were more susceptible to incubation in heather extracts from samples collected in winter compared to *T. colubriformis* eggs. The samples collected in spring also showed higher anthelmintic activity on eggs of both GIN species compared with those collected in winter, whereas when tested on *T. colubriformis* larvae, winter extracts lowered motility more than spring extracts. The inconsistency observed across the two in vitro assays could be indicative of the combination of polyphenol subgroups within these extracts interacting differently dependent on the life stage of the parasite. The association of polyphenol subgroups with egg hatching results was consistent for both GIN species, indicating that this activity was not species specific, although as *T. circumcincta* eggs were more susceptible than *T. colubriformis* in EHA*,* it appears that the interactions of these polyphenol subgroups with specific parasite proteins may be important here. This is also evident for the larval motility values as the variation that could be attributed to the polyphenol subgroups ranged from 42% for *T. circumcincta* larvae to 85% for *T. colubriformis* larvae. These results are in line with those reported in previous studies evaluating the efficacy of bioactive plant extracts on *T. colubriformis* and *T. circumcincta*, in which life stage-specific [52–54] and species-specific [55–57] susceptibility were observed.

We showed that a substantial part of the variation in anthelmintic activity was explained by changes in polyphenol subgroup content; for both life stages tested, multiple polyphenol types showed associations with the anthelmintic activity. This result supports previous evidence that polyphenol groups may interact with each other [58]. Our results are indicative of antagonistic interactions in some cases and additive effects in others. It should be noted that not all variation in anthelmintic activity can be associated with polyphenol subgroup content in the extract; furthermore, the impact on the egg hatching was not a complete reversal to the levels of the controls following incubation of extracts with PVPP. Consequently, it should be concluded that it is likely that other polyphenols not quantified here, or even non-polyphenolic compounds, are contributing to part of the anthelmintic activity observed.

While our results showed a significant impact of heather extracts on egg hatching and infective larval motility in vitro, in vivo validation will be required prior to its potential use for parasite control in small ruminant systems. Heather supplementation in goats was associated with reduced egg output and larval establishment [25] when incorporated at 30% of their diet, but only reduced faecal egg counts marginally when included in the diets of parasitised sheep at 11% of their DM intake [59]. Further investigation will be required to determine whether the active compounds present in heather will have an impact if heather is grazed by sheep. Alternatively, heather administration in the form of an extract could be another route of heather use for parasite control such as, for example, as an anthelmintic drench, as such a procedure has been demonstrated for other plants [60]. This would be particularly relevant if naturally grazed heather would not result in sufficient intake of active compounds to achieve parasite control in vivo*.*

## Conclusions

The results of this study demonstrated in vitro anthelmintic activity of heather extracts against common ovine GINs. Variation in the anthelmintic activity was associated with differences in the polyphenol subgroup contents of the extracts. Specific polyphenol subgroups within the heather extracts, such as PAs, were shown to be important when explaining variation of LD_50_ and larval motilities. However, it appears that the groups identified in the present study are not the only ones contributing to the anthelmintic effects. Additional investigation into the relationships between different groups of compounds, whether they have synergistic or antagonistic effects, would help to optimise the use of heather to reduce anthelmintic input in livestock production.

## Supplementary Information

**Additional file 1: Text S1**. RELACS: Heather sampling protocol. **Figure S1**. Collection of 10 heather samples using a “W” pattern over a 10-m^2^ area.

**Additional file 2: Table S1**. Environmental conditions at sites of heather collection.

**Additional file 3: Table S2**. Average egg hatching (%) and standard deviation (SE) for* C. vulgaris* extracts at decreasing concentrations (*n* = 3). **Table S3**. Average egg hatching (%) and SE for Spanish heather samples at decreasing concentrations (*n* = 3).

## Data Availability

A voucher specimen of the heather plant material used is stored at The Roslin Institute, Edinburgh. All data used to make conclusions are included in this published article and in the supplementary materials. The datasets used and/or analysed during the present study are available from the corresponding author upon reasonable request.

## Abbreviations

ANOVA: Analysis of variance
DMSO: Dimethyl sulfoxide
EHA: Egg hatch assay
GIN: Gastrointestinal nematode
L3: Third-stage infective larvae
LD_50_: Lethal dose for 50% inhibition
PA: Proanthocyanidin
PC: Procyanidin
PCA: Principal component analysis
PD: Prodelphinidin
PLS: Partial least squares
PBS: Phosphate-buffered saline
PSM: Plant secondary (or specialised) metabolites
PVPP: Polyvinyl polypyrrolidone
TAE: Tannic acid equivalent
UPLC-MS/MS: Ultraperformance liquid chromatography-tandem mass spectrometry
VIP: Variable importance in projection
