# Supplementary material for: Comparative analysis of the anthelmintic efficacy of European heather extracts on Teladorsagia circumcincta and Trichostrongylus colubriformis egg hatching and larval motility
Source: Parasit Vectors. 2022 Nov 4;15:409. doi: 10.1186/s13071-022-05531-0 (PMC9636748; doi:10.1186/s13071-022-05531-0)
Supplement: Supplementary file 1 — Additional file 1: Text S1. RELACS: Heather sampling protocol. Figure S1. Collection of 10 heather samples using a “W” pattern over a 10-m2 area. [file 13071_2022_5531_MOESM1_ESM.docx]

**RELACS: Heather Sampling Protocol**

WHY HEATHER

Heather species (Erica spp., Calluna vulgaris) are important vegetation components of mountainous ecosystems, mainly grazed by sheep and goats. There is anecdotal evidence of organic sheep feeding on heather plants. Heather contains condensed tannins which are known for anthelmintic activity. The hypothesis is that there will be variation in CT content and thus anthelmintic activity of heather samples around Europe. The objective in Task 4.2 is to sample and test *in vitro* the anthelmintic efficacy of heather plant extracts collected from the UK, Switzerland, Germany, Spain and Norway.

WHEN TO SAMPLE

Two sampling period are recommended, winter and spring time. Suggested samplings dates:

- November 2018 – January 2019
- March – May 2019

WHICH HEATHER SPECIES

The heather species sampled should be representative of your region, and that which is most abundant for the animals to graze on. If there are two species equally abundant that are both likely to be grazed by animals please follow this protocol for each species, collect and label the species separately. It is very important that the species are properly identified by a botanist or someone with expert knowledge, and labelled prior posting them to SRUC.

WHICH PARTS OF HEATHER

New growth, including shoots, which are available and likely to be selected by grazing animals, should be sampled. These are stands in the ‘building’ phase of growth (6 – 15 years); they are hemispherical in shape and reaching maximum height and cover. Younger plants in the ‘pioneer’ growth phase (0 – 6 years) are more pyramidal in shape and usually scattered. These may also be sampled. Older plants in the ‘mature’ phase (15 years +) are less dense in the centre, due in part to older branches collapsing sideways. These should not be sampled as the animals do not really pick those.


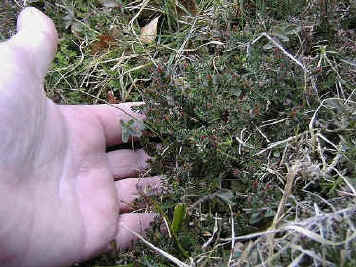

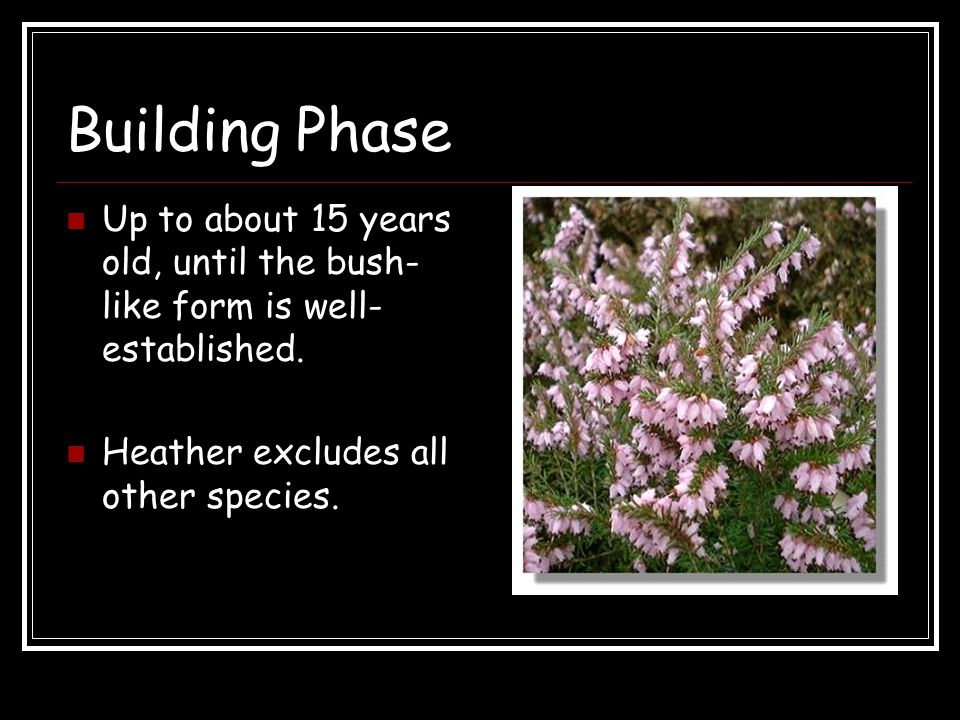


Pioneer phase Building phase

<http://www.countrysideinfo.co.uk/>

HOW TO SAMPLE

Mark out an area of 10m^2^ on the pasture using a measuring wheel. Walk through this area using a “W” pattern, sampling 10 plants throughout (Fig. 1).


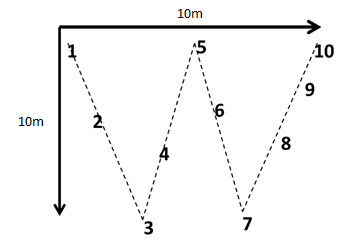


Fig. 1. Collection of 10 heather samples using a “W” pattern over 10m^2^ area

For each sampling, use garden shears to collect 50g of shoots and store them in a labelled paper bag with the following information:

- Heather species
- Institution name
- Region sampled
- Date and time sampled

SEND SAMPLES TO

Please send the samples immediately by courier to the following address:

FAO Caroline Chylinski

SRUC

Roslin Institute Building

Easter Bush, Midlothian

EH25 9RG

Scotland

*NOTE: Please do not send samples over the Christmas period e.g. 20 Dec – 2 Jan.

MATERIALS REQUIRED

Field equipment:

- Measuring wheel (or another way of measuring the sampling surface)
- Garden shears
- Paper bags / envelopes x 10
- Scales
- Permanent pen
