# Supplementary material for: Comparative analysis of the anthelmintic efficacy of European heather extracts on Teladorsagia circumcincta and Trichostrongylus colubriformis egg hatching and larval motility
Source: Parasit Vectors. 2022 Nov 4;15:409. doi: 10.1186/s13071-022-05531-0 (PMC9636748; doi:10.1186/s13071-022-05531-0)
Supplement: Supplementary file 2 — Additional file 2: Table S1. Environmental conditions at sites of heather collection. [file 13071_2022_5531_MOESM2_ESM.docx]

Table 1 - Environmental conditions at sites of heather collection.

| Site | GPS coordinates | Grazed | Weather | Shading | Site conditions |
| --- | --- | --- | --- | --- | --- |
| Castle Law, Pentland hills, Scotland, UK | 55° 51' 25.56" North (55.8705) -3° 14' 0.22" West (3.2391) | Yes | Temperature range -2 – 17℃, average 6℃. Rains 142 days/ year, rainfall 800 – 1200mm/ year. Average daylight hours 12.25  Source: NOAA | No shade | 488m above sea level,  Podzol soil. Source: themountainguide.co.uk Scottish Natural Heritage |
| Schneverdingen, Germany | 53°06'20.7"N 9°49'04.1"E | No | Temperature range 18.6 – 1.6℃, average 9.9℃. Average rainfall 822mm. Daylight hours 2.32 – 9.93. Source: meteorological site at Soltau. | No shade | 92m above sea level, Podzol soil. |
| Smøla, Norway | 63°18’2.771’’ North (63.3007697) 8°5’47.139’’ East (8.0964275) | Yes | Average temp 7.9℃ from 2004-2020; drought in spring, temporary snow cover in winter. Rainfall 1080 – 1540mm/year, average 1276mm/year. Daylight hours. 4.5 – 20.5 Source: timeanddate.no | No shade | 15m above sea level, close to ocean so formed from moraine and sea erosion. Soil is peat over sand and gravel from sea erosion of morainic sediments. Source: Geological survey of Norway. |
| Oberer Hummel, Switzerland | 47°05’42.72’’ North 8°46’23.52’’ East | Yes | Cool, wet, snow cover in winter (Nov – March), Average temperature 6.25℃. Average rainfall 1380mm/ year. Average daylight hours 12.15 (8.5 – 16 range).  Source: Bestereisezeit.ch | No shade | 1,250 – 1,350m above sea level. Heavy, humus-poor loamy soils, pH 5. |
| Illano, Asturias, Spain | 43°19'33.12" North (43.325886), 6°53'40.18" West (-6.894498) | Yes | Temperature range 1.38 – 19.98℃, average temperature 9.78℃; humid. Average rainfall 1561mm, only 100mm/month between October and April. Average daylight hours 13 hours 52 minutes. Source: meteorological station at field site. | No shade | 800 – 1000m above sea level, acidic and nutrient poor leptosols soil. |
