# Supplementary material for: Comparative analysis of the anthelmintic efficacy of European heather extracts on Teladorsagia circumcincta and Trichostrongylus colubriformis egg hatching and larval motility
Source: Parasit Vectors. 2022 Nov 4;15:409. doi: 10.1186/s13071-022-05531-0 (PMC9636748; doi:10.1186/s13071-022-05531-0)
Supplement: Supplementary file 3 — Additional file 3: Table S2. Average egg hatching (%) and standard deviation (SE) for C. vulgaris extracts at decreasing concentrations (n = 3). Table S3. Average egg hatching (%) and SE for Spanish heather samples at decreasing concentrations (n = 3). [file 13071_2022_5531_MOESM3_ESM.docx]

Table S2 – Average egg hatching (%) and S.E. for C. vulgaris extracts at decreasing concentrations (n=3).

| GIN species | Season | Country | Concentration (mg/ml) | Egg hatching (%) | S.E. |
| --- | --- | --- | --- | --- | --- |
| *T. circumcincta* | Winter | UK | 10 | 0.00 | 0.00 |
|  |  |  | 5 | 1.50 | 1.50 |
|  |  |  | 2.5 | 63.3 | 8.15 |
|  |  |  | 1.25 | 88.5 | 4.58 |
|  |  |  | 0.625 | 98.8 | 1.16 |
|  | Spring |  | 10 | 0.00 | 0.00 |
|  |  |  | 5 | 0.00 | 0.00 |
|  |  |  | 2.5 | 59.0 | 2.47 |
|  |  |  | 1.25 | 97.5 | 0.09 |
|  |  |  | 0.625 | 99.3 | 0.66 |
|  | Winter | Germany | 10 | 2.95 | 1.82 |
|  |  |  | 5 | 48.5 | 18.3 |
|  |  |  | 2.5 | 95.9 | 0.94 |
|  |  |  | 1.25 | 91.5 | 2.47 |
|  |  |  | 0.625 | 100 | 0.00 |
|  | Spring |  | 10 | 1.60 | 0.80 |
|  |  |  | 5 | 0.81 | 0.81 |
|  |  |  | 2.5 | 66.7 | 10.1 |
|  |  |  | 1.25 | 98.8 | 0.62 |
|  |  |  | 0.625 | 94.7 | 0.80 |
|  | Winter | Norway | 10 | 0.00 | 0.00 |
|  |  |  | 5 | 80.7 | 4.20 |
|  |  |  | 2.5 | 96.0 | 1.56 |
|  |  |  | 1.25 | 98.9 | 1.06 |
|  |  |  | 0.625 | 95.9 | 2.54 |
|  | Spring |  | 10 | 4.96 | 2.67 |
|  |  |  | 5 | 5.10 | 2.81 |
|  |  |  | 2.5 | 89.4 | 0.91 |
|  |  |  | 1.25 | 94.2 | 1.55 |
|  |  |  | 0.625 | 98.4 | 0.82 |
|  | Winter | Switzerland | 10 | 2.79 | 2.79 |
|  |  |  | 5 | 3.88 | 2.23 |
|  |  |  | 2.5 | 83.3 | 5.78 |
|  |  |  | 1.25 | 87.4 | 5.46 |
|  |  |  | 0.625 | 92.4 | 3.63 |
|  | Spring |  | 10 | 1.00 | 1.00 |
|  |  |  | 5 | 4.29 | 1.43 |
|  |  |  | 2.5 | 97.1 | 1.93 |
|  |  |  | 1.25 | 98.6 | 0.71 |
|  |  |  | 0.625 | 100 | 0.00 |
|  | Winter | Spain | 10 | 0.00 | 0.00 |
|  |  |  | 5 | 1.31 | 0.65 |
|  |  |  | 2.5 | 77.9 | 2.22 |
|  |  |  | 1.25 | 95.0 | 2.90 |
|  |  |  | 0.625 | 96.7 | 1.67 |
|  | Spring |  | 10 | 0.00 | 0.00 |
|  |  |  | 5 | 3.08 | 0.80 |
|  |  |  | 2.5 | 51.2 | 7.07 |
|  |  |  | 1.25 | 96.0 | 0.56 |
|  |  |  | 0.625 | 97.7 | 1.14 |
| *T. colubriformis* | Winter | UK | 10 | 0.00 | 0.00 |
|  |  |  | 5 | 12.2 | 1.76 |
|  |  |  | 2.5 | 96.0 | 2.00 |
|  |  |  | 1.25 | 99.3 | 0.66 |
|  |  |  | 0.625 | 100 | 0.00 |
|  | Spring |  | 10 | 0.00 | 0.00 |
|  |  |  | 5 | 0.00 | 0.00 |
|  |  |  | 2.5 | 32.7 | 10.0 |
|  |  |  | 1.25 | 95.6 | 0.71 |
|  |  |  | 0.625 | 98.8 | 0.65 |
|  | Winter | Germany | 10 | 5.10 | 3.34 |
|  |  |  | 5 | 95.7 | 2.15 |
|  |  |  | 2.5 | 96.7 | 0.41 |
|  |  |  | 1.25 | 99.1 | 0.90 |
|  |  |  | 0.625 | 99.0 | 1.00 |
|  | Spring |  | 10 | 0.68 | 0.68 |
|  |  |  | 5 | 0.68 | 0.68 |
|  |  |  | 2.5 | 77.4 | 5.81 |
|  |  |  | 1.25 | 92.1 | 1.72 |
|  |  |  | 0.625 | 99.6 | 0.43 |
|  | Winter | Norway | 10 | 27.6 | 5.85 |
|  |  |  | 5 | 95.6 | 3.19 |
|  |  |  | 2.5 | 95.4 | 2.37 |
|  |  |  | 1.25 | 100 | 0.00 |
|  |  |  | 0.625 | 96.3 | 0.32 |
|  | Spring |  | 10 | 0.68 | 0.68 |
|  |  |  | 5 | 10.0 | 4.84 |
|  |  |  | 2.5 | 90.3 | 5.65 |
|  |  |  | 1.25 | 96.5 | 1.94 |
|  |  |  | 0.625 | 98.7 | 0.68 |
|  | Winter | Switzerland | 10 | 12.9 | 4.87 |
|  |  |  | 5 | 47.5 | 6.31 |
|  |  |  | 2.5 | 98.9 | 0.56 |
|  |  |  | 1.25 | 97.6 | 1.36 |
|  |  |  | 0.625 | 98.6 | 0.82 |
|  | Spring |  | 10 | 0.00 | 0.00 |
|  |  |  | 5 | 37.9 | 23.0 |
|  |  |  | 2.5 | 86.2 | 2.89 |
|  |  |  | 1.25 | 99.2 | 0.84 |
|  |  |  | 0.625 | 97.1 | 1.51 |
|  | Winter | Spain | 10 | 0.97 | 0.97 |
|  |  |  | 5 | 3.28 | 1.15 |
|  |  |  | 2.5 | 97.4 | 1.11 |
|  |  |  | 1.25 | 100 | 0.00 |
|  |  |  | 0.625 | 99.1 | 0.90 |
|  | Spring |  | 10 | 1.82 | 0.91 |
|  |  |  | 5 | 1.70 | 0.14 |
|  |  |  | 2.5 | 55.1 | 9.39 |
|  |  |  | 1.25 | 96.2 | 1.06 |
|  |  |  | 0.625 | 98.0 | 1.05 |
| Significance (P values) | | |  |  |  |
| GIN species | | <0.001 |  |  |  |
| Season | | <0.001 |  |  |  |
| Country | | <0.001 |  |  |  |
| Concentration | | <0.001 |  |  |  |
| GIN species x season | | <0.001 |  |  |  |
| GIN species x country | | 0.014 |  |  |  |
| GIN species x concentration | | <0.001 |  |  |  |
| Season x country | | <0.001 |  |  |  |
| Season x concentration | | <0.001 |  |  |  |
| Country x concentration | | <0.001 |  |  |  |
| GIN species x season x country | | 0.131 |  |  |  |
| GIN species x season x concentration | | 0.006 |  |  |  |
| GIN species x country x concentration | | <0.001 |  |  |  |
| Season x country x concentration | | <0.001 |  |  |  |
| Country x GIN species x season x concentration | | <0.001 |  |  |  |

Table S3 - Average egg hatching (%) for Spanish heather samples at decreasing concentrations (n=3).

| GIN species | Season | Heather species | Concentration (mg/ml) | Egg hatching (%) | S.E. |
| --- | --- | --- | --- | --- | --- |
| *T. circumcincta* | Winter | *Calluna vulgaris* | 10 | 0.00 | 0.00 |
|  |  |  | 5 | 1.31 | 0.65 |
|  |  |  | 2.5 | 77.9 | 2.22 |
|  |  |  | 1.25 | 95.0 | 2.90 |
|  |  |  | 0.625 | 96.7 | 1.67 |
|  | Spring |  | 10 | 0.00 | 0.00 |
|  |  |  | 5 | 3.08 | 0.80 |
|  |  |  | 2.5 | 51.2 | 7.07 |
|  |  |  | 1.25 | 96.0 | 0.56 |
|  |  |  | 0.625 | 97.7 | 1.14 |
|  | Winter | *Erica cinerea* | 10 | 0.00 | 0.00 |
|  |  |  | 5 | 4.82 | 0.43 |
|  |  |  | 2.5 | 84.1 | 4.54 |
|  |  |  | 1.25 | 97.0 | 1.69 |
|  |  |  | 0.625 | 95.9 | 2.25 |
|  | Spring |  | 10 | 0.00 | 0.00 |
|  |  |  | 5 | 6.10 | 1.85 |
|  |  |  | 2.5 | 92.4 | 1.44 |
|  |  |  | 1.25 | 96.4 | 2.97 |
|  |  |  | 0.625 | 96.3 | 3.70 |
| *T. colubriformis* | Winter | *Calluna vulgaris* | 10 | 0.97 | 0.97 |
|  |  |  | 5 | 3.28 | 1.15 |
|  |  |  | 2.5 | 97.4 | 1.11 |
|  |  |  | 1.25 | 100 | 0.00 |
|  |  |  | 0.625 | 99.1 | 0.90 |
|  | Spring |  | 10 | 1.82 | 0.91 |
|  |  |  | 5 | 1.70 | 0.14 |
|  |  |  | 2.5 | 55.1 | 9.39 |
|  |  |  | 1.25 | 96.2 | 1.06 |
|  |  |  | 0.625 | 98.0 | 1.05 |
|  | Winter | *Erica cinerea* | 10 | 4.78 | 2.98 |
|  |  |  | 5 | 55.1 | 7.04 |
|  |  |  | 2.5 | 96.5 | 2.63 |
|  |  |  | 1.25 | 96.8 | 0.75 |
|  |  |  | 0.625 | 96.0 | 1.24 |
|  | Spring |  | 10 | 0.00 | 0.00 |
|  |  |  | 5 | 5.99 | 0.27 |
|  |  |  | 2.5 | 86.6 | 4.59 |
|  |  |  | 1.25 | 97.4 | 1.79 |
|  |  |  | 0.625 | 97.7 | 1.44 |
| Significance (P values) | | |  |  |  |
| GIN species | | < 0.001 |  |  |  |
| Season | | < 0.001 |  |  |  |
| Heather species | | <0.001 |  |  |  |
| Concentration | | <0.001 |  |  |  |
| GIN species x season | | < 0.001 |  |  |  |
| GIN species x heather species | | 0.104 |  |  |  |
| GIN species x concentration | | < 0.001 |  |  |  |
| Season x heather species | | 0.295 |  |  |  |
| Season x concentration | | < 0.001 |  |  |  |
| Heather species x concentration | | < 0.001 |  |  |  |
| GIN species x season x concentration | | < 0.001 |  |  |  |
| GIN species x heather species x concentration | | < 0.001 |  |  |  |
| GIN species x heather species x season | | 0.012 |  |  |  |
| Heather species x concentration x season | | < 0.001 |  |  |  |
| Heather species x GIN species x concentration x season | | < 0.001 |  |  |  |
